# Both exogenous 5-HT and endogenous 5-HT, released by fluoxetine, enhance distension evoked propulsion in guinea-pig ileum *in vitro*

**DOI:** 10.3389/fnins.2014.00301

**Published:** 2014-09-19

**Authors:** Rachel M. Gwynne, Amanda J. Clarke, John B. Furness, Joel C. Bornstein

**Affiliations:** ^1^Department of Physiology, University of MelbourneParkville, VIC, Australia; ^2^Departments of Anatomy and Cell Biology, University of MelbourneParkville, VIC, Australia

**Keywords:** serotonin, 5-HT_3_ receptors, 5-HT_4_ receptors, NK_3_ tachykinin receptors, intestinal motility

## Abstract

The roles of 5-HT_3_ and 5-HT_4_ receptors in the modulation of intestinal propulsion by luminal application of 5-HT and augmentation of endogenous 5-HT effects were studied in segments of guinea-pig ileum *in vitro*. Persistent propulsive contractions evoked by saline distension were examined using a modified Trendelenburg method. When 5-HT (30 nM), fluoxetine (selective serotonin reuptake inhibitor; 1 nM), 2-methyl-5-HT (5-HT_3_ receptor agonist; 1 mM), or RS 67506 (5-HT_4_ receptor agonist, 1 μM) was infused into the lumen, the pressure needed to initiate persistent propulsive activity fell significantly. A specific 5-HT_4_ receptor antagonist, SB 207266 (10 nM in lumen), abolished the effects of 5-HT, fluoxetine, and RS 67506, but not those of 2-methyl-5-HT. Granisetron (5-HT_3_ receptor antagonist; 1 μM in lumen) abolished the effect of 5-HT, fluoxetine, RS 67506, and 2-methyl-5-HT. The NK_3_ receptor antagonist SR 142801 (100 nM in lumen) blocked the effects of 5-HT, fluoxetine, and 2-methyl-5-HT. SB 207266, granisetron, and SR 142801 had no effect by themselves. Higher concentrations of fluoxetine (100 and 300 nM) and RS 67506 (3 and 10 μM) had no effect on the distension threshold for propulsive contractions. These results indicate that luminal application of exogenous 5-HT, or increased release of endogenous mucosal 5-HT above basal levels, acts to lower the threshold for propulsive contractions in the guinea-pig ileum via activation of 5-HT_3_ and 5-HT_4_ receptors and the release of tachykinins. The results further indicate that basal release of 5-HT is insufficient to alter the threshold for propulsive motor activity.

## Introduction

The intestinal mucosa is the major site of synthesis of serotonin (5-HT) in the body, but the role of mucosal 5-HT in controlling intestinal motility remains controversial. Gastric emptying, jejunal transit and transit through the entire gastrointestinal tract *in vivo* are normal in mice lacking tryptophan hydroxylase 1, the rate limiting enzyme for 5-HT synthesis in enterochromaffin (EC) cells (Li et al., 2011). However, *in vitro* analysis of propulsive motility in the colon from the same mice indicates that 5-HT released by EC cells facilitates normal propulsion; in knock out animals, reflex responses to distension were reduced and only larger fecal pellets were propelled (Heredia et al., 2013). These studies are consistent with other studies showing that complete removal of the mucosa and blockade of mucosal 5-HT release does not prevent initiation of colonic propulsion (Spencer et al., 2011), colonic contractile complexes (Keating and Spencer, 2010) or polarized reflex responses of the muscle (Costa and Furness, 1976). This may reflect the finding that distension does not produce 5-HT release from the colonic mucosa (Grider et al., 1996). However, luminal application of 5-HT antagonists can slow, but not prevent, propulsion in guinea-pig colon (Jin et al., 1999). Thus, it appears that colonic propulsion does not require mucosal 5-HT, but may be enhanced when mucosal 5-HT is released.

A similar picture is seen in the small intestine where mucosal 5-HT does not initiate propulsive motor activity evoked by distension, but may facilitate this activity (Tuladhar et al., 1997). Moreover, the mechanisms by which mucosally released 5-HT activates different motor patterns in the small intestine of the guinea-pig appear to be complex. Mucosal 5-HT plays a key role in nutrient (decanoic acid) induced segmentation in guinea-pig small intestine as blocking either 5-HT_3_ or 5-HT_4_ receptors, at the level of the mucosa, virtually abolishes this motor pattern (Ellis et al., 2013). However, while luminal decanoic acid causes an increase in both segmenting and propulsive motor activity, luminal fluoxetine (100–300 nM) which releases mucosal 5-HT only triggers segmenting activity, again via 5-HT_3_ and 5-HT_4_ receptors (Ellis et al., 2013). On the other hand, cholera toxin, which would be expected to release mucosal 5-HT, increases propulsive motor activity at resting intraluminal pressures and enhances propulsive motor patterns evoked by distension, each via mechanism(s) independent of 5-HT_3_ receptors (Fung et al., 2010).

It has been reported that propulsive motor activity evoked by saline distension is enhanced by luminal 5-HT via 5-HT_3_ receptors close to, if not in, the mucosal epithelium and that 5-HT_4_ receptors are not required (Tuladhar et al., 1997). In contrast, local inhibitory reflexes evoked by amino acids transiently applied to the mucosa are depressed by blockade of both 5-HT_3_ and 5-HT_4_ receptors, but not blockade of either subtype on its own (Gwynne and Bornstein, 2007). Furthermore, 5-HT applied via the serosa can enhance saline evoked propulsive motor patterns via 5-HT_4_ receptors, while EC cells have been shown to express 5-HT_4_ receptors (Hoffman et al., 2012). Whether 5-HT_3_ and 5-HT_4_ mediated effects of exogenous 5-HT are independent of each other or are part of the same pathway is unclear, as is their relevance to the roles of endogenous 5-HT. This study was designed to address these issues.

Electrophysiological studies of guinea-pig ileum indicate that 5-HT applied to the mucosa excites the mucosal terminals of AH neurons with cell bodies in the myenteric plexus via 5-HT_3_ receptors (Bertrand et al., 1997, 2000; Bertrand and Bornstein, 2002). Mucosally applied 5-HT also evokes slow excitatory synaptic potentials (EPSPs) in myenteric AH neurons (Bertrand et al., 1997, 2000) presumably via synapses from directly activated AH neurons. These slow EPSPs would be expected to increase the firing of distension-sensitive AH neurons, thus enhancing reflexes evoked by distension. Most slow EPSPs in myenteric AH neurons are blocked by the specific NK_3_ tachykinin receptor antagonist SR 142801 (Alex et al., 2001; Johnson and Bornstein, 2004) which suggests that luminally applied 5-HT may facilitate propulsive reflexes in the guinea-pig ileum via the release of a tachykinin. Accordingly, we also investigated this possibility.

Propulsive motor activity was studied using a modified Trendelenberg preparation in which the stimulus was a saline distension; agonists and antagonists were added to the luminal perfusion solution. The concentration of 5-HT used was 30 nM as preliminary experiments indicated that this was sufficient to facilitate propulsive reflexes. The time of exposure was over 1 h to mimic the effects of a nutrient stimulus that might release 5-HT (Gwynne et al., 2004; Ellis et al., 2013). The effects of exogenous 5-HT were compared with those of endogenous 5-HT whose effects were revealed by a selective serotonin reuptake inhibitor, fluoxetine.

## Materials and methods

### Tissue preparation

Guinea-pigs (150–350 g) of either sex were killed by being stunned and having their carotid arteries severed. This procedure was approved by the University of Melbourne Animal Experimentation Ethics Committee. The composition (in mM) of physiological saline used in all experiments was: NaCl 118, KCl 4.8, NaH_2_PO_4_ 1, NaHCO_3_ 25, MgSO_4_ 1.2, d-glucose 11, CaCl_2_ 2.5, bubbled with 95% O_2_, 5% CO_2_. Segments of ileum 8–10 cm in length, were taken 10–20 cm proximal to the ileo-caecal junction, flushed clean, cannulated, and placed in an organ bath containing physiological saline at 37°C. Intraluminal pressure was measured via a T-piece connected to the anal cannula and recorded using a BIOPAC Systems MP100 recording unit and Acknowledge v. 3.25 software (SDR Clinical technology, NSW). In some experiments, video images of the intestinal segment were recorded and later processed using edge-detection software to produce spatio-temporal maps. These methods have previously been described in Gwynne et al. (2004) and confirmed that the recorded pressure increases corresponded to a propulsive motor pattern.

### Experimental protocols

Once the ileal segments had been dissected and cannulated, 10 mL of the physiological saline was flushed through the lumen and the preparation left to equilibrate for 1 h. During the equilibration period, the height of the saline in the reservoir was level with the segment of ileum, so the inflow pressure head was held to zero, below the threshold for initiation of propulsive contractions. Following equilibration, the pressure threshold for initiation of persistent propulsive contractions (measured as repeated transient increases in intraluminal pressure, see Gwynne et al., 2004 for definition) was determined by raising the inflow pressure in steps of 1 cm H_2_O at intervals of approximately 30 s, until this motor pattern was initiated. The inflow pressure was then returned to control levels. The pressure which triggered the persistent propulsive contractions was consistent for several hours in any one preparation and was taken as the threshold for propulsive motor activity (or peristalsis) (Gwynne et al., 2004). After each trial, the height of the inflow reservoir was returned to the starting level and the preparation was allowed to rest for 10 min. After 3–4 control measurements, an agonist (or fluoxetine) was added to the inflow reservoir and the drug was flushed into the lumen. The preparation was allowed to equilibrate for 1 h before a second set of 3 threshold measurements was recorded. When the effects of antagonists were studied after the initial exposure to an agonist, approximately 30 mL of fresh saline was flushed through the lumen and left to equilibrate for 20 min before a combination of agonist and antagonist was applied luminally. After a further 1 h equilibration period, a final set of 3 threshold measurements was made. Each antagonist was also tested by itself using the protocol for agonists. Where video recordings were made, images were acquired at a frame rate of 10 frames per second for the duration of the threshold measurement.

Data in the text is given as mean ± s.e.m. except where otherwise stated.

Statistical comparisons were made using paired *t*-tests or One Way analysis of variance (ANOVA); *P*-values < 0.05 were considered significant.

### Drugs

Drugs used were 5-HT, fluoxetine, 2-methyl-5-hydroxytryptamine (2-me-5-HT) (all from Sigma Aldrich, NSW), RS 67506 (Tocris Cookson Ltd. Bristol, UK), tetrodotoxin (Alomone Labs Ltd. Jerusalem, Israel), granisetron, SB 207266 (both supplied by Glaxo SmithKline, Harlow, UK), and SR 142801 (supplied courtesy of Dr. X. Emonds-Alt, Sanofi Recherche, France). All were initially made up in distilled water to make stock solutions. To avoid oxidation, new 5-HT solutions were made up each week.

## Results

**Figure 1:**
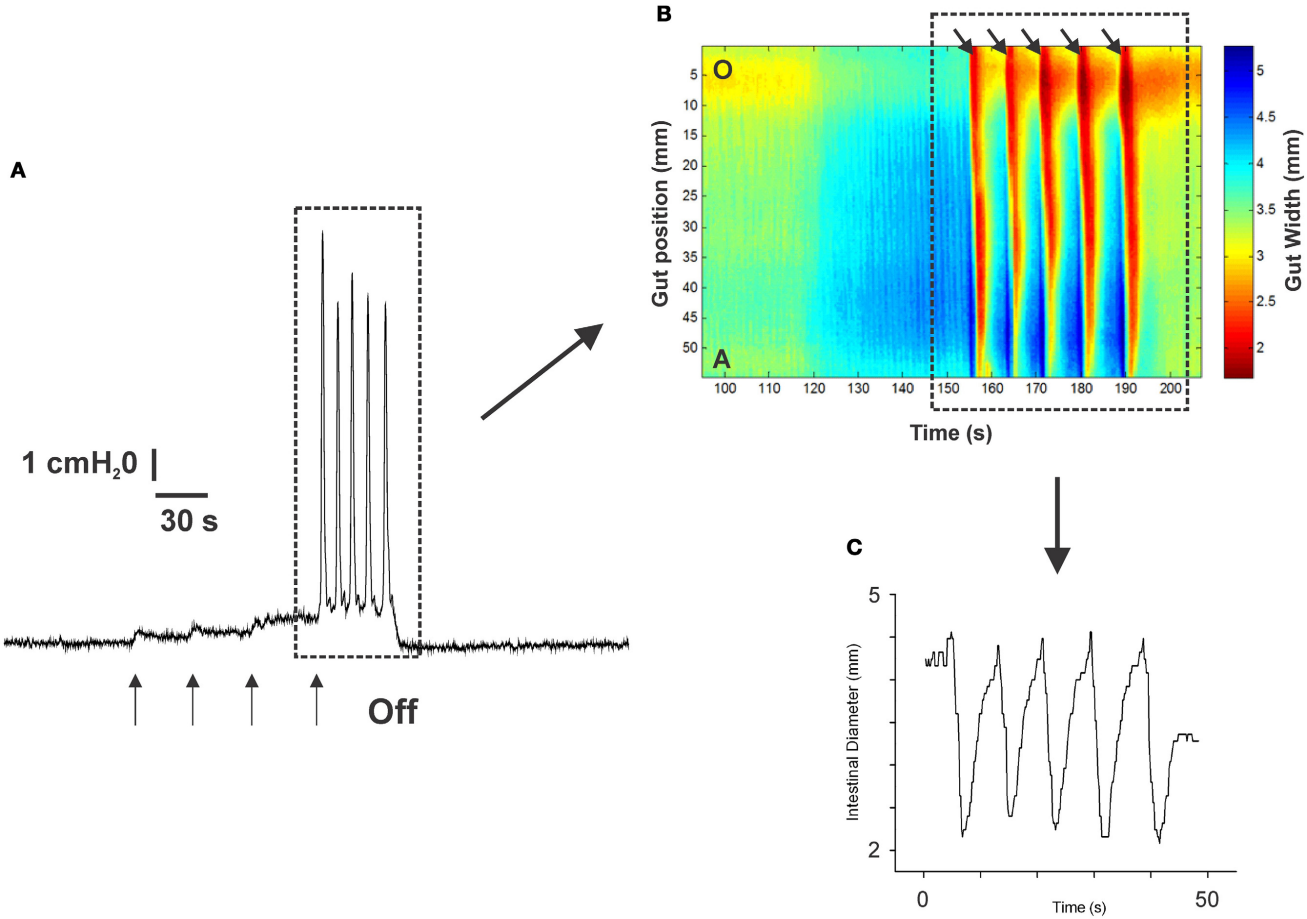
**The propulsive motor pattern (peristalsis) in an isolated segment of guinea-pig ileum**. The segment was distended in cumulative 1 cm H_2_O steps by raising the lumenal perfusion reservoir, which caused phasic increases in intraluminal pressure **(A)**. **(B)** The corresponding spatio-temporal map of the propulsive contractions (black arrows) recorded at threshold. Contraction is shown in red and dilation in blue. **(C)** The diameter changes occurring at one point along the intestinal segment during the propulsive contractions (see dotted black line in **B**) at threshold. The pressure at which this motor pattern was initiated was highly reproducible in any one preparation.

**Figure 2:**
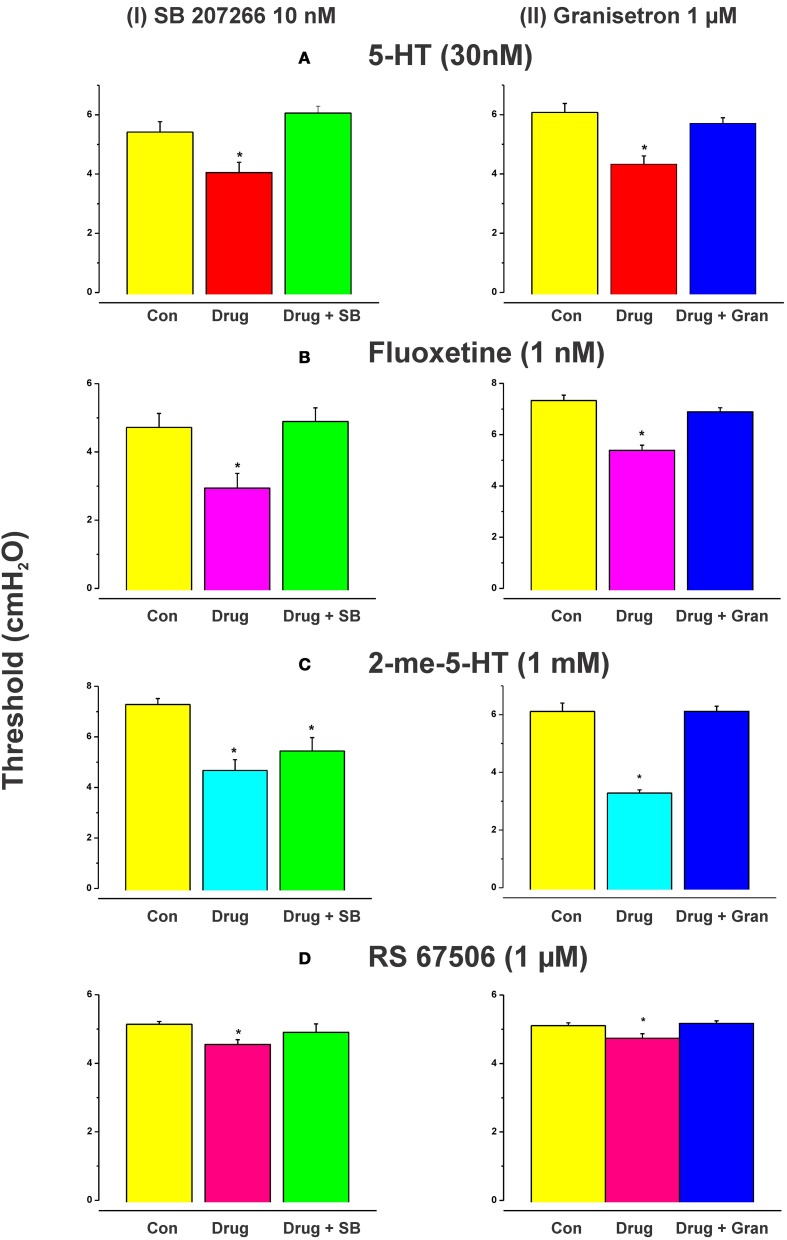
**Effects of exogenous 5-HT, endogenous 5-HT potentiated by fluoxetine, 2-me-5-HT, and RS 67506 on the threshold pressure for initiation of propulsive contractions**. Histograms showing the mean (±s.e.m.) thresholds in cm H_2_O for initiation of peristalsis in control, with the drug present or with drug and antagonist present. 5-HT (30 nM), fluoxetine (1 nM), 2-me-5-HT (1 mM), and RS 67506 (1 μM) each reversibly reduced the threshold for initiation of propulsive motor patterns in the ileum when applied to the lumen. The reduction in threshold was significant in each case [**(A)** 5-HT, *P* < 0.01; **(B)** fluoxetine, *P* < 0.01, 2-me-5-HT, *P* < 0.0001, RS 67506, *P* < 0.01]. The histograms in the left column show the effects of the 5-HT_4_ receptor antagonist SB 207266 (10 nM) in the lumenal perfusate on the changes in threshold for initiation of propulsive motor patterns in the ileum induced by 5-HT **(A)**, fluoxetine **(B)**, 2-me-5-HT **(C)**, or RS 67506 **(D)**. The right column shows the effects of the 5-HT_3_ receptor antagonist granisetron (1 μM) on the changes induced by the same compounds. SB 207266 blocked the facilitation caused by luminal 5-HT, fluoxetine, and RS 67506 [panels **A(I),B(I),D(I)**] but did not abolish the effects of 2-me-5-HT [panel **C(I)**]. In contrast, granisetron blocked the facilitation of peristalsis produced by all of the agonists tested [**A(II),B(II),C(II),D(II)**]. ^*^indicates significantly different from control with *P* < 0.05 in all cases.

Figure 1 shows an example of the propulsive contractions evoked by increasing the intraluminal pressure to threshold. Step-wise increases in intraluminal pressure to levels below the threshold for persistent propulsive contractions sometimes evoked one or more propagating contractions of the circular muscle (not shown here). We have previously described these as “transient propulsive contractions” (Gwynne et al., 2004). Threshold pressures that evoked consistent propulsive contractions ranged from 4 to 8 cm H_2_O (median 6.5 cm H_2_O) in 102 preparations. When the threshold was reached, the propulsive contractions were regular and persisted throughout the period of increased luminal pressure, ceasing immediately the pressure was reduced below the threshold (Figure 1). Luminal 5-HT (30 nM) and luminal fluoxetine (1 nM) reduced the threshold for the persistent propulsive contractions by 25 and 40%, respectively (Figures 2A,B; 5-HT −*P* < 0.01, *n* = 6, fluoxetine −*P* < 0.001, *n* = 6). In each case, the effects reversed on washout of the agonist. The changes in threshold were accompanied by a statistically significant increase in the rate of propagation of the propulsive contractions measured via spatiotemporal maps (control 21.6 ± 1.1 mm/s, 5-HT 30.4 ± 1.4 mm/s, *n* = 6, *P* < 0.001; control 14.5 ± 1.0 mm/s, fluoxetine 20.2 ± 1.3 mm/s, *n* = 4, *P* < 0.01) and a significant reduction in the diameter of the ileum at the threshold pressure (control 3.2 ± 0.02 mm, 5-HT 2.8 ± 0.01 mm, *P* < 0.05, *n* = 4; control 3.0 ± 0.02 mm, fluoxetine 2.4 ± 0.01 mm, *P* < 0.05, *n* = 4).

Higher concentrations of fluoxetine (100 and 300 nM) in the lumen had no significant effect on the threshold pressure for the persistent propulsive contractions (control 4.8 ± 0.1 100 nM fluoxetine 4.8 ± 0.1 cm H_2_O; control 4.6 ± 0.1, 300 nM fluoxetine 4.7 ± 0.1 cm H_2_O) although they did produce an increase in contractile activity at basal pressures. The effects of luminal 5-HT were mimicked by the specific 5-HT_3_ receptor agonist 2-me-5-HT (1 mM, *n* = 6), and by a specific 5-HT_4_ receptor agonist RS 67506 (1 μM, *n* = 6), which reduced the pressure threshold for propulsive contractions by 35% (*P* < 0.0001) and 12% (*P* < 0.01), respectively (Figures 2C,D). As with fluoxetine, higher concentrations of RS 67506 (3 or 10 μM) produced no apparent change in threshold for activation of persistent propulsive contractions, but increased the overall contractile activity at basal pressures. In each case, the increased contractile activity at basal pressures involved predominantly segmenting contractions (see Figure 1 of Ellis et al., 2013).

When added to the luminal perfusion solution, a highly specific 5-HT_4_ receptor antagonist SB 207266 (10 nM) (Wardle et al., 1996) blocked the facilitation caused by luminal 5-HT, fluoxetine, and RS 67506 [Figures 2A(I),B(I),D(I), all *n* = 6] but did not alter the effects of 2-me-5-HT [Figure 2C(I), *n* = 6]. In contrast, a specific 5-HT_3_ receptor antagonist granisetron (1 μM) (29) blocked the facilitation of persistent propulsive contractions produced by all agonists tested. (5-HT *n* = 8, fluoxetine *n* = 6, RS 67506 *n* = 6 and 2-me-5-HT *n* = 6) [Figures 2A(II),B(II),C(II),D(II)].

SB 207266 (10 nM) and granisetron (1 μM) when added to the luminal perfusate on their own, or in combination, had no effect on the threshold pressure for initiation of persistent propulsive contractions (**Figure 4**, *n* = 6 in each case).

### Role of NK_3_ tachykinin receptors

**Figure 3:**
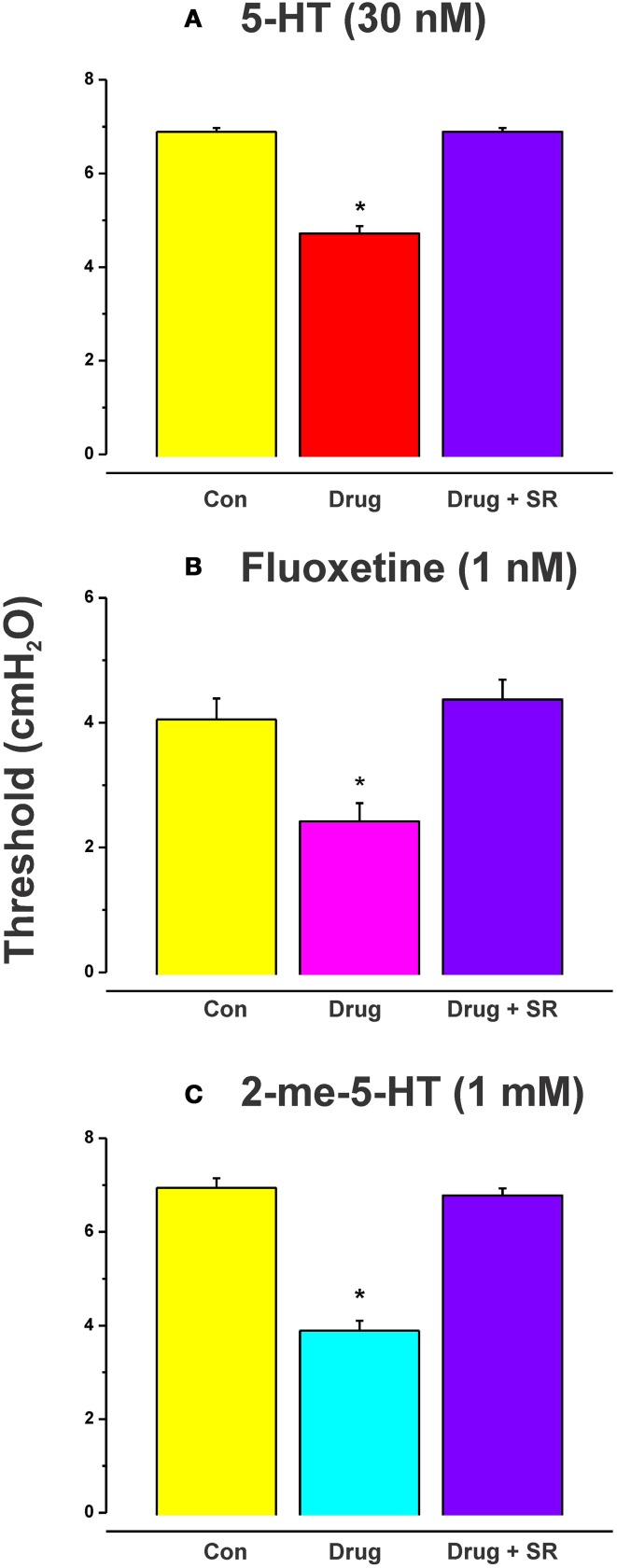
**Effects of blockade of NK_3_ tachykinin receptors on the reduction in threshold for initiation of propulsion evoked by serotonin agonists**. The specific NK_3_ tachykinin receptor antagonist SR 142801 (SR; 100 nM) blocked the reduction in threshold for initiation of propulsive contractions in the ileum induced by 5-HT **(A)**, fluoxetine **(B)**, and 2-me-5-HT **(C)**. ^*^Indicates significantly different from control with *P* < 0.05 in all cases.

**Figure 4:**
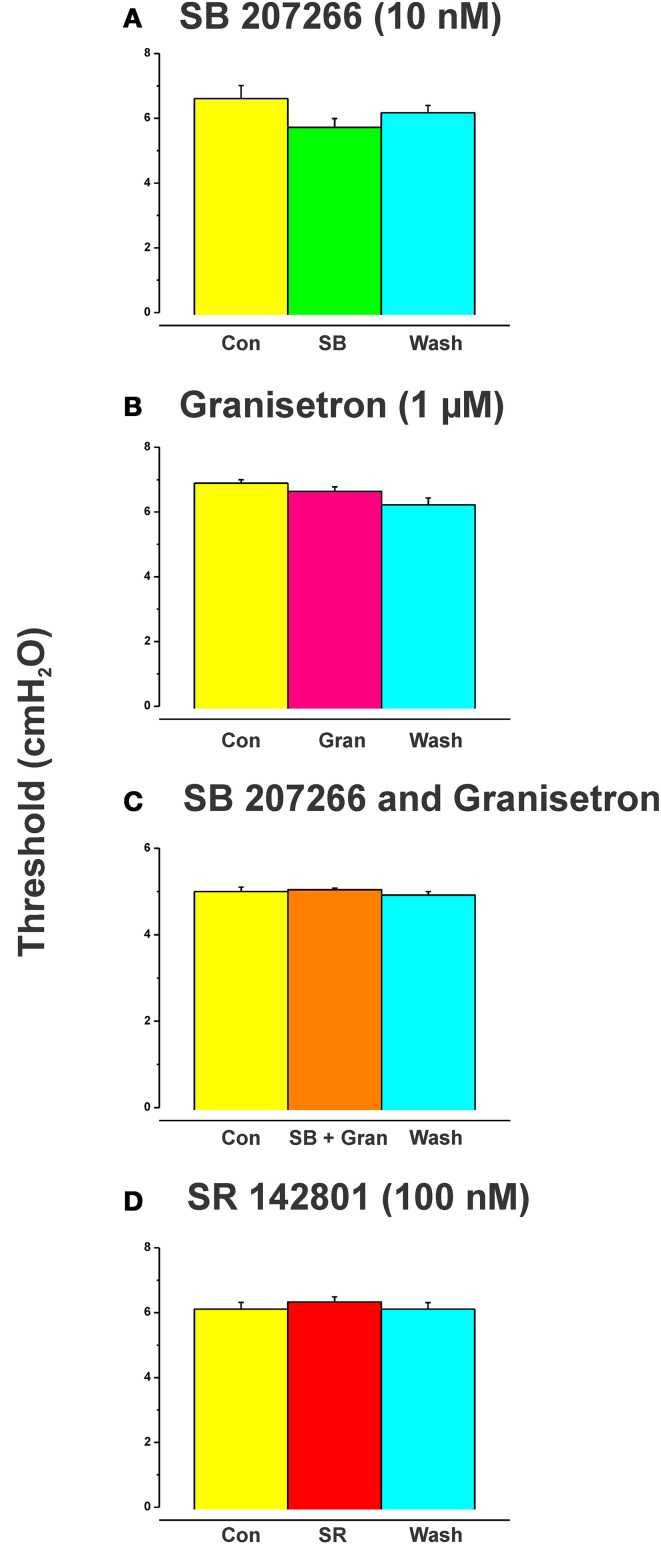
**Effects of blockade of 5-HT_4_, 5-HT_3_, and NK_3_ receptors on the threshold for initiation of propulsion in the absence of other drugs**. SB 207266 and granisetron when added to the luminal perfusate on their own, or in combination, had no effect on the threshold pressure for initiation of propulsive contractions **(A–C)**. The specific NK_3_ tackykinin receptor antagonist SR 142801 (100 nM) also had no effect on the threshold pressure for propulsive contractions **(D)**.

The effects of luminal 5-HT, fluoxetine, and 2-me-5HT were all prevented by simultaneous addition of the specific NK_3_ tachykinin receptor antagonist SR 142801 (100 nM) to the luminal perfusate (Figure 3, each *n* = 6, RS 67506 not tested). SR 142801 had no effect on the threshold pressure when added to the luminal perfusate on its own (Figure 4D, *n* = 6) or when added to the superfusing solution (not illustrated).

## Discussion

This study indicates that perfusion of exogenous 5-HT through the lumen, or increases in release of endogenous mucosal 5-HT above basal levels, facilitates intestinal propulsion evoked by saline distension of the ileum via activation of 5-HT_4_ and 5-HT_3_ receptors and the release of tachykinins acting at NK_3_ receptors. We found no evidence that basal release of mucosal 5-HT was sufficient to activate these receptors.

### Site of action of 5-HT

**Figure 5:**
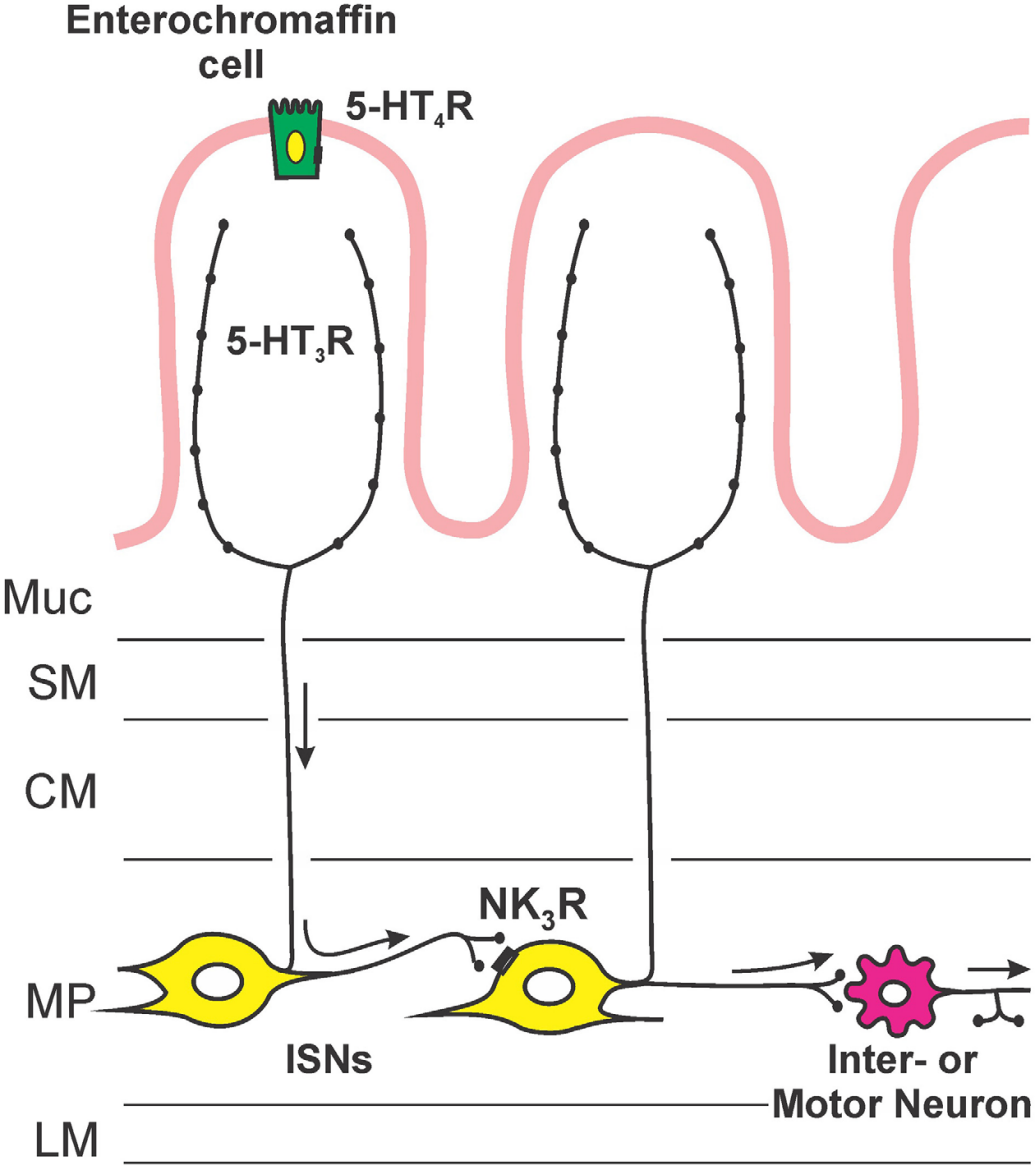
**An hypothesis about the sequence of mechanisms by which exogenous or endogenous 5-HT in the lumen enhances propulsion in the guinea-pig ileum**. 5-HT acts on 5-HT_4_ receptors on enterochromaffin cells to release more 5-HT, this higher concentration of 5-HT then excites mucosal terminals of myenteric AH neurons via 5-HT_3_ receptors. The myenteric AH neurons then release tachykinins to enhance the excitability of distension-sensitive myenteric AH neurons via NK_3_ receptors.

Figure 5 illustrates the sites of action and locations of receptors that are deduced from this study. Facilitation of propulsion by 5-HT or fluoxetine was reversed by either the 5-HT_4_ receptor antagonist, SB 207266 or the 5-HT_3_ receptor antagonist, granisetron. Furthermore, luminal application of the specific 5-HT_3_ receptor agonist 2-me-5-HT or 5-HT_4_ receptor agonist RS 67506 mimicked the effects of 5-HT and fluoxetine. This indicates that the mechanism underlying the facilitation occurs via activation of both 5-HT_3_ and 5-HT_4_ receptors in this system. These data contrast with previous research by Tuladhar et al. (1997) who found that mucosally applied 5-HT facilitates propulsive reflexes via activation of 5-HT_3_ receptors, but they found no evidence for 5-HT_4_ receptor involvement. One reason for this difference is likely to be the marked differences in concentration of 5-HT used in the two studies. We observed facilitation with 30 nM 5-HT, three orders of magnitude lower than the lowest effective concentration used by Tuladhar et al. (1997). Previous studies have indicated that 5-HT_3_ receptors in guinea-pig ileum are unlikely to be directly activated by 30 nM 5-HT. For example, Lucchelli et al. (1995) found that at least 300 nM 5-HT is needed to produce minimal activation of 5-HT_3_ receptors on neurons in this tissue. Thus, the concentrations used by Tuladhar et al. would directly excite 5-HT_3_ receptors in a manner analogous to 2-me-5-HT in the present study, but 30 nM 5-HT might only activate the receptors indirectly, for example by releasing mucosal 5-HT. The initial action of luminal 5-HT is probably at a receptor with a much higher affinity for 5-HT. The EC_50_ for 5-HT_4_ receptors in this tissue is 15 nM (Buchheit et al., 1985), which is consistent with these receptors being the targets of low concentrations of 5-HT applied in the lumen. This was demonstrated directly in the present study, since SB 207266 blocked the effects of both 5-HT and RS 67506. Interestingly, granisetron reversed the facilitation induced by both RS 67506 and 2-me-5-HT, whereas SB 207266 had no effect on the facilitation induced by 2-me-5-HT. That is, 5-HT_3_ receptors are required when 5-HT_4_ receptors facilitate peristalsis, but not the reverse. This implies that the two types of receptor are arranged in series with 5-HT_4_ receptors preceding 5-HT_3_ receptors in the pathway (Figure 5). Both classes of 5-HT receptors are widely expressed within the enteric neural circuitry, but the efficacy of the luminally applied antagonists suggests that the receptors for facilitation of propulsion are in, or close, to the mucosa. Evidence for this conclusion comes from the finding that these antagonists have differing effects on motility when they are applied to the lumen or to the serosa in modified Trendelenberg preparations (Tuladhar et al., 1997; Ellis et al., 2013). The most likely mechanism is that low concentrations of 5-HT in the lumen act via 5-HT_4_ receptors on EC cells to release enough 5-HT to raise the mucosal concentration to a level capable of exciting 5-HT_3_ receptors (Figure 5). Guinea-pig EC cells are immunoreactive for 5-HT_4_ receptors and activation of these receptors leads to release of 5-HT (Hoffman et al., 2012). The 5-HT_3_ receptors involved in the facilitation of propulsion by luminal 5-HT are probably those on mucosal nerve terminals of myenteric AH neurons that have been found to be excited by mucosal application of 5-HT (Bertrand et al., 2000).

### Role of NK_3_ tachykinin receptors

The facilitation of peristalsis induced by 5-HT, fluoxetine, and 2-me-5-HT was blocked by the specific NK_3_ tachykinin antagonist, SR 142801. This indicates that the release of a tachykinin, and its subsequent interaction with NK_3_ receptors, plays a critical role in the facilitatory pathway. NK_3_ tachykinin receptors are found on many neurons in the guinea-pig enteric nervous system, including myenteric AH neurons (Jenkinson et al., 1999) and are implicated in both ascending and descending reflex pathways in the guinea-pig ileum (Johnson et al., 1996, 1998). However, their role in reflexes in dissected flat sheet preparations is largely confined to responses to mucosal distortion, while reflex responses to distension are unaffected by blockade of these receptors (Johnson et al., 1998). This suggests that NK_3_ receptors are involved in the circuitry underlying the facilitation of distension by luminal 5-HT, but not in reflex pathways activated by distension. Slow EPSPs mediated by NK_3_ tachykinin receptors are prominent in myenteric AH neurons (Alex et al., 2001; Johnson and Bornstein, 2004). Furthermore, computer simulations show that slow synaptic transmission within the circumferentially projecting network of connections between myenteric AH neurons is important for determining the magnitudes of responses to ongoing physiological stimuli (Thomas et al., 2000, 2004). This raises the possibility that excitation of myenteric AH neurons by luminal 5-HT leads to activation of a recurrent feedback circuit involving slow transmission (via NK_3_ receptors) between myenteric AH neurons. This could in turn generate enhanced responsiveness to sensory stimuli such as distension, i.e., facilitation of propulsion.

This leads us to postulate that low concentrations of 5-HT act on 5-HT_4_ receptors on EC cells in the mucosa, releasing 5-HT that then activates 5-HT_3_ receptors on mucosal terminals of myenteric AH neurons. Higher concentrations of 5-HT in the lumen (Tuladhar et al., 1997) or 5-HT_3_ agonists like 2-me-5-HT act directly on the mucosal nerve endings of myenteric AH neurons, bypassing the requirement for activation of 5-HT_4_ receptors. The activation of myenteric AH neurons leads to the release of tachykinins acting at NK_3_ receptors on other myenteric AH neurons, thereby enhancing their overall excitability and making them more sensitive to the saline distensions used to excite peristalsis (Figure 4).

### Mechanism of action of fluoxetine

The similar sensitivity of responses to 5-HT_4_ antagonists, and the fact that fluoxetine is a specific inhibitor of 5-HT reuptake, suggests that 1 nM fluoxetine acts to increase the extracellular concentration of 5-HT in the intestinal mucosa enough to activate 5-HT_4_ receptors in a similar way to 30 nM 5-HT. This could also account for the enhancement by 1 nM fluoxetine of propulsion of artificial fecal pellets in the guinea-pig distal colon (Wade et al., 1996). However, higher concentrations of fluoxetine (100 and 300 nM) had no effect on the initiation of propulsive motor patterns, and Wade et al. (1996) found that such concentrations of fluoxetine inhibited colonic transit (a result we have confirmed in an unpublished study). Thus, while low concentrations of fluoxetine facilitate propulsive motor patterns in both ileum and colon, higher concentrations do not.

The concentration dependence may result from the properties of the 5-HT transporter itself. Chen et al. (1998) found that the *K*_i_ for fluoxetine on a cloned guinea-pig serotonin transporter was about 48 nM. That is, fluoxetine depresses reuptake of 5-HT at concentrations comparable to those that inhibit propulsive motor patterns in the colon and has very little effect at concentrations that facilitate ileal or colonic transit. Thus, increases in extracellular 5-HT produced by low concentrations of fluoxetine may be highly localized, with uninhibited transporters limiting spread of 5-HT beyond the vicinity of the mucosal terminals of enteric neurons. At higher concentrations, transporter capacity might be reduced sufficiently to allow diffusion of 5-HT to more distant sites where it can interact with inhibitory 5-HT_1A_ receptors (Galligan et al., 1988), thereby suppressing the facilitation of peristalsis or even reversing it.

### Role of mucosal 5-HT

There is no doubt that release of mucosal 5-HT plays a major role in the nausea and vomiting produced by ingestion of various toxins (Sanger and Andrews, 2006) and the hypersecretion leading to diarrhea produced by luminal exposure to either the bacterium *Vibrio cholera* or its exotoxin, cholera toxin (Farthing, 2002; Lundgren, 2002). However, other physiological roles of mucosal 5-HT are significantly less clear. The present data strongly indicate that mucosal 5-HT is not necessary for distension to evoke propulsive motor activity. Both the absence of an effect of luminal 5-HT_3_ or 5-HT_4_ antagonists on their own on the threshold pressure for distension-evoked propulsion and previous results showing that such reflexes can be evoked in the absence of the mucosa (Costa and Furness, 1976; Yokoyama and Ozaki, 1980; Tsuji et al., 1992; Spencer et al., 2011) or mucosal 5-HT (Heredia et al., 2013) support this conclusion. This contrasts with the finding that nutrient-induced segmentation is largely suppressed by 5-HT_3_ or 5-HT_4_ antagonists in the lumen of the small intestine (Ellis et al., 2013). Both physiological data and computer simulations suggest that the initial sensory transduction step in nutrient-induced segmentation is mediated by both cholecystokinin (CCK) and 5-HT, with mucosal 5-HT also playing a role in providing feedback to enter neural motor pattern generators about the overall contractile state of the muscle (Chambers et al., 2011; Ellis et al., 2013). It has recently been demonstrated that a specific subset of enteroendocrine cells contains both CCK and 5-HT, while there are others that contain only one of these mediators (Cho et al., 2014). Thus, the physiological role of mucosal 5-HT may vary according to the stimulus that evokes its release. Chemical stimuli, different nutrients, and other food components, might be expected to produce moderate levels of release in various combinations with other mucosal mediators, while contractile activity appears to release 5-HT via mechanical deformation of the mucosa (Bertrand, 2006).

## Conclusions

This study proposes a way in which 5-HT facilitates propulsive contractions in the guinea-pig small intestine. Increased availability of 5-HT at low concentrations in the mucosa of the intestine activates high affinity 5-HT_4_ receptors, which triggers the release of more 5-HT. This then acts on 5-HT_3_ receptors to excite enteric neural circuits, likely to involve release of tachykinins that facilitate propulsive reflexes.

### Conflict of interest statement

The authors declare that the research was conducted in the absence of any commercial or financial relationships that could be construed as a potential conflict of interest.
